# Association of Time in Target Range of Resting Heart Rate With Adverse Clinical Outcomes in Patients With Acute Coronary Syndromes After Percutaneous Coronary Intervention

**DOI:** 10.5334/gh.1384

**Published:** 2025-01-17

**Authors:** Jianmei Zheng, Cen Chen, Zhongcai Fan, Qiang Ye, Yi Zhong, Jinsong Li, Hao Huang, Jianping Deng, Jinghong Zhao, Tinglin Xiong, Wenjie Tian, Xuemei Zhang

**Affiliations:** 1 The Affiliated Hospital of Southwest Medical University, Luzhou, China; 2 The First People’s Hospital of Chongqing Liang Jiang New Area, Chongqing, China; 3 Sichuan Academy of Medical Sciences & Sichuan Provincial People’s Hospital, Chengdu, China; 4 Nanchong Central Hospital, The Second Clinical Medical College, North Sichuan Medical College, Nanchong, China

**Keywords:** Acute Coronary Syndromes, percutaneous coronary intervention, heart rate, clinical outcomes

## Abstract

Heart rate (HR) has been proved to be associated with major adverse cardiovascular events (MACE) in Acute coronary syndrome patients. However, the threshold value and clinical significance of time in target of resting heart rate (TTR-HR) remain insufficiently elucidated. Our study aimed to evaluate the independent association between TTR-HR and cardiovascular outcomes in the follow-up study of ACS. A total of 1455 ACS patients who underwent percutaneous coronary intervention (PCI) and were admitted to 22 hospitals between 2019 and 2022 were enrolled and followed up for 12 months. MACE was defined as a composite of cardiac death, nonfatal recurrent myocardial infarction, ischemic-driven revascularization, and ischemic stroke. The association between TTR-HR and cardiovascular outcomes was assessed using Cox regression model. Compared to patients with TTR-HR 0–50% and >50%–75%, patients with TTR-HR > 75%–100% were older and less alcohol user, less likely to use diuretics and anti-diabetic drugs, these patients had less comorbidities of hyperlipidemia, diabetes, heart failure, and cardiac shock. After 12 months follow up, the incidence of MACE and composite endpoint but not mortality was higher in patients with TTR-HR 0–50% and >50%–75% than those with TTR-HR > 75%–100%. After multivariate adjustment, TTR-HR [hazard ratio = 2.11, 95% CI: 1.19–3.74, p = 0.01] was independently associated with composite endpoint. In summary, our study demonstrates that TTR-HR holds significant prognostic value, with TTR-HR > 75%–100% being independently associated with reduced composite endpoint risk in ACS patients following PCI. These findings emphasize the importance of effective heart rate control in ACS patients following PCI.

## 1 Introduction

Acute coronary syndrome (ACS) is a serious cardiovascular disease characterized by a sudden decrease in blood supply to the heart, encompassing non-ST elevation myocardial infarction (NSTEMI), and ST elevation myocardial infarction (STEMI), which poses a significant burden on global healthcare (1,2). According to statistics, an estimated more than seven million people in the world are diagnosed with ACS every year, and the incidence of ACS in China is also increasing year by year (1). Fortunately, PCI has revolutionized the treatment of ACS over the past decade, improving prognosis and reducing clinical event rates and mortality (3). While these advances have improved clinical outcomes, patients with ACS still face high risks for clinical events, such as recurrent myocardial infarction, heart failure, and death.

Heart rate is a crucial factor affecting the clinical outcome of ACS patients undergoing PCI, even for those who require heart rate control, which can alleviate cardiovascular symptoms, improve hemodynamics, and reduce the incidence of clinical events (4). Elevated heart rate exacerbates myocardial ischemia and is associated with an increased risk of adverse clinical outcomes in ACS patients (5). Accordingly, heart rate reduction with ivabradine or beta-blockers could improve myocardial blood flow and systolic function in ischemic myocardium, alleviate cardiac remodeling and improve clinical outcomes in patients with symptomatic heart failure (6). While the significance of heart rate in ACS management is acknowledged, the specific impact of heart rate control on clinical outcomes in ACS patients undergoing PCI is still an area of ongoing research. Furthermore, studies on heart rate typically assess the average heart rate or a single measurement value during the observation period to determine the impact of heart rate on clinical outcomes. This overlooks the fact that heart rate is a constantly changing vital sign. Effective heart rate management should consider the heart rate level and the duration of maintaining that level. However, the impact of the duration of arrhythmia on ACS patients undergoing PCI is not yet clear. Therefore, more research is needed to understand the duration of maintaining heart rate level affects the health of ACS patients undergoing PCI, which can help improve treatment effectiveness and clinical outcomes.

This study aims to investigate the association of heart rate control with adverse clinical outcomes, such as MACE and deaths, in ACS patients undergoing PCI. By elucidating the impact of heart rate control on clinical outcomes in this specific patient population, this research has the potential to inform clinical practice and optimize the management of ACS patients undergoing PCI.

## 2 Methods

### 2.1 Study design and population

This study is a retrospective analysis utilizing data extracted from Center for Digital Management and Follow-up of cardiovascular Diseases, HeartMed, (Chengdu, Sichuan, China) that was conducted at four clinical sites. The study protocol was approved by HEARTMED Digital Management System at each site and all participants provided informed consent. Review the data of coronary heart disease patients who participated in digital management and received continuous follow-up from September 2019 to December 2022. The diagnosis criteria for coronary heart disease (CHD) refer to those formulated by the World Health Organization (WHO). Patients are enrolled in a digital management system for long-term follow-up through digital systems or telephone, and the follow-up data is promptly recorded in the database. Inclusion criteria: (1) Patients with coronary heart disease undergoing PCI; (2) All patients had an ECG showing sinus rhythm; (3) Enrolled in the Center for Digital Management and Follow-up of cardiovascular Diseases, HeartMed with a follow-up time of 12 months. And exclusion criteria: (1) Patients whose hospital admission diagnosis does not include ACS disease; (2) Patients with missing heart rate data.

### 2.2 Data collection

From the database, retrospective analysis was used to extract and summarize clinical data of patients with a follow-up of 12 months. The following parameters were noted: demographics (including: age, gender, alcohol consumption history and smoking history), vital signs (blood pressure and heart rate), family history (including history of hypertension, coronary heart disease, diabetes mellitus or stroke), and accompanying illnesses (including hypertension, hyperlipidemia, diabetes, hyperuricemia, stroke, heart failure, anemia, cardiogenic shock, chronic kidney disease). In addition, we have also collected information about the medications prescribed upon discharge (β-blockers, ACEI/ARB, calcium channel blockers, diuretics, statins, PCSK9 inhibitors, aspirin, antiplatelet agents, antianginal agents, PPIs, antidiabetic drugs) and relevant laboratory results during hospitalization, including LDL-C, Creatinine, Hemoglobin, Platelets, AST, ALT, Uric Acid, LVEF and HbA1c. Additionally, Clinical outcomes were investigated through telephone interview with patients and/or medical records review, and all data is cross-checked by two researchers.

### 2.3 Time in target range of resting heart rate

Resting heart rate was obtained by the Bioland blood pressure tester(Bioland, A223) for 1 minute after the patient had been sitting quietly for ≥5 minutes. All patients were asked to measure blood pressure and heart rate at 8 am and 8 pm every day, at least four measurements per month were included in the study. Traditional heart rate control only considers the single value or average of resting heart rate, which is not sufficient to assess the objective status of heart rate and its impact on clinical outcomes. The optimal indicators for heart rate management should consider the level of heart rate, its stability, and the time of reaching the expected level. This article selects time in target range (TTR) of resting heart rate to evaluate the level of heart rate control (7,8). This method adds each patient’s time within the target range (≤80 bpm per minute) and divides it by the total time of observation. This assumes that between-measurement heart rate varies linearly. TTR of resting heart rate is divided into three groups (0–50% > 50%–75% > 75%–100%) based on this criterion.

### 2.4 Ascertainment of clinical outcome

The primary outcome was the composite endpoint: death from any cause, non-fatal myocardial infarction, angina, or hospitalization for heart failure. Secondary endpoints were the individual components including MACE and death from any cause. MACE included non-fatal myocardial infarction, angina, or hospitalization for heart failure at 12 months. Study physicians independently assessed all MACE using protocol-specified endpoint definitions based on a review of relevant medical records (9). This study utilized the database of HeartMed Digital Management System, the organization that provided the data collected by the study.

### 2.5 Statistical analysis

Statistical analysis was performed using SPSS (SPSS 22.0, IBM Corp., Armonk, NY, USA). Continuous variables are presented as mean±standard deviation (SD) and were compared using the independent student’s t-test (If the analyzed data conforms to the normal distribution), otherwise, as median and interquartile ranges (IQRs), and compared using the Mann-Whitney U test. Categorical variables are presented as frequency and percentage and were compared by Pearson’s chi-squared test or Fisher’s exact test (if an expected cell count of the contingency table was fewer than five). Kaplan-Meier method was used to plot the 12-month curve of ACS patients without endpoint events, and log-rank test was used to estimate equality of event-free survival (10). The Univariate and multi factorial Cox regression risk models were utilized to estimate hazard ratios (HR) and 95% confidence intervals (95% CI) for intergroup comparison of clinical outcomes. In all analyses, P-values < 0.05 were considered statistically significant.

## 3 Results

### 3.1 Baseline characteristics

**Figure 1:**
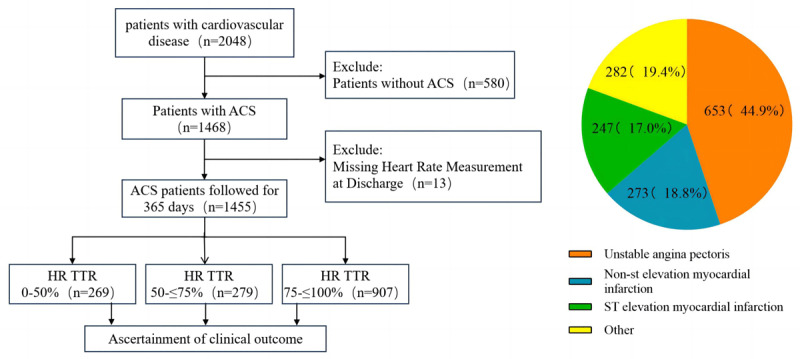
Composition chart of cardiovascular disease patients.

Cardiovascular patient data (n = 2048) from September 2019 to December 2022 were derived from the “HEARTMED Digital Management System” database, 580 non-ACS patients (28.3%) and 13 patients(0.6%) with missing heart rate data were excluded, and 1455 ACS patients were finally divided into three groups, TTR-HR 0–50% (n = 269), TTR-HR > 50%–75% (n = 279) and TTR-HR > 75%–100% (n = 907) (Figure 1). Among the 1455 ACS patients, the mean age of the study population was 62.3 ± 10.9 years, including 1119 males (76.9%) and 336 females (23.1%). There were statistically significant differences between the three groups in age, smoking, diuretics, Statins, aspirin, hypoglycemic drugs, hyperlipidemia, diabetes, stroke, heart failure, cardiogenic shock, systolic blood pressure, diastolic blood pressure, and heart rate (P < 0.05), while other parameters were not significant different between the three groups(all > 0.05), as shown in Table 1.

**Table 1:** Characteristics of participants according to TTR-HR.


OBSERVATION ITEM	0–50% (n = 269)	>50%–75% (n = 279)	>75%–100% (n = 907)	TOTAL (n = 1455)	P

Age	60.7 ± 11.1	60.3 ± 11.6	63.1 ± 10.5	62.3 ± 10.9	**<0.001**

Male, n(%)	202(75.1)	228(81.7)	689(76.0)	1119(76.9)	0.100

Smoking, n(%)	114(42.5)	150(53.8)	409(45.2)	673(46.4)	**0.017**

Drinking, n(%)	155(57.8)	152(54.5)	477(52.8)	784(54.0)	0.339

Hypertension, n(%)	24(9.0)	29(10.4)	90(10.0)	143(9.9)	0.848

History of Heart disease, n(%)	17(6.3)	15(5.4)	64(7.1)	96(6.6)	0.586

History of diabetes, n(%)	16(6.0)	13(4.7)	45(5.0)	74(5.1)	0.762

History of stroke, n(%)	4(1.5)	2(0.7)	11(1.2)	17(1.2)	0.648

β-blocker, n(%)	179(66.8)	196(70.3)	655(72.3)	1030(70.9)	0.215

ACEI/ARB, n(%)	154(57.5)	176(63.1)	589(65.0)	919(63.2)	0.080

Calcium channel blocker, n(%)	57(21.3)	54(19.4)	211(23.3)	322(22.2)	0.353

Diuretic, n(%)	86(32.1)	73(26.2)	199(22.0)	358(24.6)	**0.003**

Statins, n(%)	267(99.6)	271(97.1)	896(98.9)	1434(98.7)	**0.032**

PCSK9, n(%)	17(6.3)	20(7.2)	68(7.5)	105(7.2)	0.812

Ezetimibe, n(%)	71(26.5)	72(25.8)	207(22.8)	350(24.1)	0.358

Aspirin, n(%)	201(77.5)	232(83.2)	796(87.9)	1229(84.6)	**<0.001**

P2Y12 inhibitor, n(%)	267(99.6)	276(98.9)	903(99.8)	1446(99.6)	0.169

Antianginal drugs, n(%)	35(13.1)	37(13.3)	115(12.7)	187(12.9)	0.968

PPI, n(%)	132(49.3)	116(41.6)	425(46.9)	673(46.3)	0.169

Hypoglycemic drug, n(%)	129(48.1)	102(36.6)	274(30.2)	505(34.8)	**<0.001**

Hypertension, n(%)	166(61.7)	162(58.1)	563(62.1)	891(61.2)	0.482

Hyperlipidemia, n(%)	108(40.1)	103(36.9)	284(31.3)	495(34)	**0.015**

Diabetes, n(%)	112(41.6)	95(34.1)	254(28.0)	461(31.7)	**<0.001**

Hyperuricemia, n(%)	30(11.2)	36(12.9)	84(9.3)	150(10.3)	0.194

Stroke, n(%)	29(10.8)	32(11.5)	144(15.9)	205(14.1)	**0.041**

Heart failure, n(%)	79(29.4)	48(17.2)	129(14.2)	256(17.6)	**<0.001**

Anemia, n(%)	19(7.1)	14(5.0)	46(5.1)	79(5.4)	0.425

Cardiogenic shock, n(%)	21(7.8)	20(7.2)	22(2.4)	63(4.3)	**<0.001**

Chronic kidney disease, n(%)	15(5.6)	25(9.0)	52(5.7)	92(6.3)	0.132

UA, umol/L	326.6(274.0, 380.0)	358.0(304.0, 430.6)	341.7(281.9, 418.0)	343(279.0, 420.1)	0.608

Ast, u/L	23(18.6, 29.0)	25(20.8, 31.2)	24(19.05, 29.5)	24(19.8, 29.6)	0.447

Alt, u/L	24(16.9, 38.9)	29.5(21.0, 41.4)	27.9(19.3, 38.0)	27.6(19.2, 39.0)	0.219

Plt, 109/L	215(182.5, 245.5)	213(161.5, 245.5)	192(171.0, 235.0)	199(171.8, 238.0)	0.232

Ldlc, mmol/L	1.8(1.5, 2.4)	1.82(1.2, 2.2)	1.655(1.3, 2.0)	1.7(1.3, 2.1)	0.139

Crea, umol/L	74.8(66, 88.6)	77.5(69.15, 96)	71.9(62.0, 88.0)	74(63.0, 88.6)	0.180

Hbalc, %	7.0(6.6, 7.3)	6.0(5.8, 6.0)	6.9(6.3, 7.4)	6.8(6.0, 7.4)	0.337

Hb, g/L	136.4 ± 19	144.1 ± 14.5	139.6 ± 15.6	140.0 ± 16.0	0.090

LVEF, %	62.8 ± 6.6	59.4 ± 11.6	62.1 ± 7.9	61.8 ± 8.4	0.571

Target blood pressure, n(%)	209(77.7)	210(75.3)	662(73.0)	1081(74.3)	0.279

Systolic pressure, mmHg	121(110,134)	124(113,137)	124(123,138)	123(112, 137)	0.008

Diastolic pressure, mmHg	77(70,84)	76(69,85)	75(68,82)	76(68,83)	0.005

Heart rate, per minute	85(78,92)	79(72,86)	72.0(66.0,80.0)	76(68,85)	<0.001

Weight, kg	70(60.5,79)	70(64,77.5)	71.0(64.0, 80.0)	70.(63.7,80.0)	0.381

BMI	26.6(22.85,30.05)	26.7(23.5,29.7)	27.1(23.9,30.1)	26.8(23.7,30.0)	0.289


### 3.2 Potential predictors of major clinical adverse events

**Figure 2:**
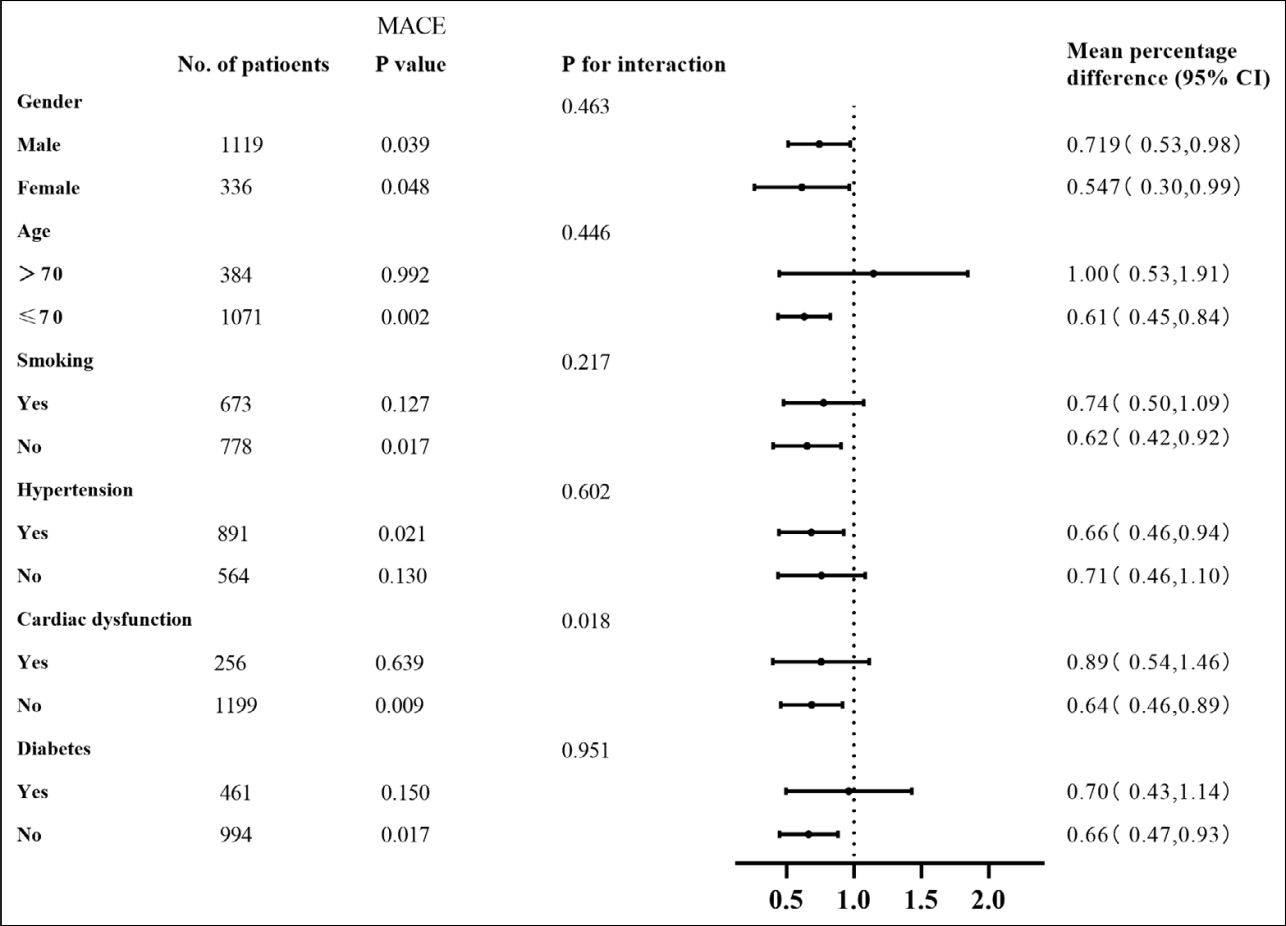
Hazard ratio for cardiovascular outcomes.

In adjusted analyses across key subgroups of interest, univariate COX regression showed that history of hypertension (HR: 2.29, 95% CI: 1.25–4.20, P = 0.007), heart failure (HR: 1.88, 95% CI: 1.11–3.20, P = 0.019), diuretic (HR: 2.25, 95% CI: 1.38–3.65, P = 0.001,) and arrhythmia (HR: 0.51, 95% CI: 0.31–0.82, P = 0.005) had statistically significant effects on cumulative adverse events. After adjusting for various confounding factors, the multivariate regression analysis showed that history of hypertension (HR 2.4, 95% CI: 1.30–4.45, P = 0.005), diuretic (HR 2.02, 95% CI: 1.12–3.65, P = 0.019) and arrhythmia (HR 0.54, 95% CI: 0.33–0.87, P = 0.012) were still a significant correlation with cumulative adverse events in ACS patients. In total subjects, TTR-HR > 75% is an independent protective factor for clinical adverse events in ACS. Table 2 summarizes the results of the COX regression analysis. When stratified by gender, age, smoking statues, hypertension and diabetes, a consistent pattern of association was observed between higher HR TTR and lower MACE risk (Figure 2). Meanwhile, the interaction between HR TTR with heart failure was statistically significant (P < 0.05).

**Table 2:** Cox regression analysis of single and multiple factors related to adverse events.


OBSERVATION ITEM	SINGLE FACTORS			MULTIPLE FACTORS		
	
	HR	95%CI	P	HR	95%CI	P

Age	0.99	0.97–1.01	0.192			

male	0.80	0.44–1.47	0.470			

Smoking	1.35	0.84–2.19	0.216	1.29	0.79–2.09	0.307

Drinking	1.19	0.73–1.93	0.487			

History of hypertension	2.29	1.25–4.20	**0.007**	2.43	1.31–4.49	**0.005**

History of Heart disease	1.66	0.76–3.64	0.203			

History of diabetes	1.20	0.44–3.28	0.730			

Hypertension	0.88	0.54–1.4	0.596			

Hyperlipidemia	0.95	0.57–1.58	0.841			

Diabetes	0.99	0.59–1.66	0.969			

Hyperuricemia	1.73	0.91–3.30	0.096			

Apoplexy	0.70	0.32–1.54	0.379			

Heart failure	1.88	1.11–3.20	**0.019**	1.16	0.60–2.24	0.660

History of stent implantation surgery	0.95	0.57–1.59	0.856			

Anemia	1.75	0.76–4.05	0.191			

Cardiogenic shock	2.26	0.98–5.23	0.057			

Chronic kidney disease	1.20	0.48–2.99	0.693			

β-blocker	1.57	0.87–2.83	0.133	**1.56**	0.86–2.82	0.141

ACEI/ARB	0.85	0.52–1.39	0.521			

CCB	1.10	0.63–1.93	0.740			

Diuretic	2.25	1.38–3.65	**0.001**	1.99	1.10–3.58	**0.022**

PCSK9	1.29	0.56–2.98	0.555			

Ezetimibe	1.35	0.80–2.28	0.259			

Aspirin	0.92	0.48–1.76	0.810			

Antianginal drugs	1.20	0.61–2.34	0.603			

PPI	1.20	0.74–1.94	0.455			

Hypoglycemics	1.28	0.78–2.08	0.325			

arrhythmia	0.51	0.31–0.82	**0.005**	0.52	0.32–0.85	**0.010**

Target blood pressure	1.02	0.59–1.77	0.938			


### 3.3 Associations of time in target range and cardiovascular outcomes

As shown in Table 3, compared to the incidence of composite endpoint (3.3%) in the highest TTR-HR group (>75%–100%), the other two groups (>50%–75%, 0–50%) had a significant association with the incidence of composite endpoint (6.8%, 6.7%, p = 0.010). compared to the incedence of major adverse cardiovascular events(3.0%) in the highest TTR-HR group (>75%–100%), the other two groups (>50%–75%, 0–50%) had a significant association with the incidence of major adverse cardiovascular events(5.4%, 5.9%, p = 0.036). Nevertheless, TTR-HR was not associated with death events in the three heart rate range groups (Table 3).

**Table 3:** The associations between heart rate response and composite endpoint and MACE.


OBSERVATION ITEM	0–50% (n = 269)	>50%–75% (n = 279)	(>75%–100%) (n = 907)	TOTAL (n = 1455)	P

Composite endpoint, n(%)	18(6.7)	19(6.8)	30(3.3)	67(4.6)	**0.010**

MACE, n(%)	16(5.9)	15(5.4)	27(3.0)	58(4.0)	**0.036**

Death events, n(%)	3(1.1)	4(1.4)	3(0.3)	10(0.7)	0.078


### 3.4 Association between TTR-HR with risk of cumulative adverse events

**Figure 3:**
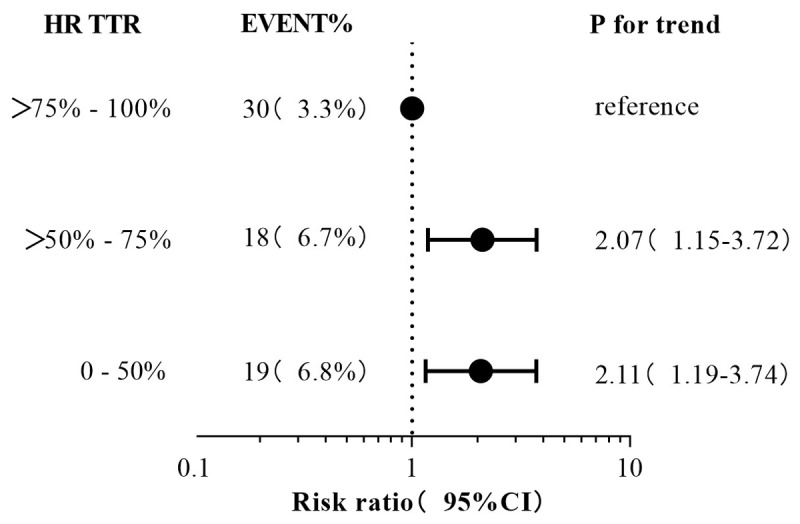
Pre-specified subgroups analyses of the association between heart rate in target range and composite endpoint.

**Figure 4:**
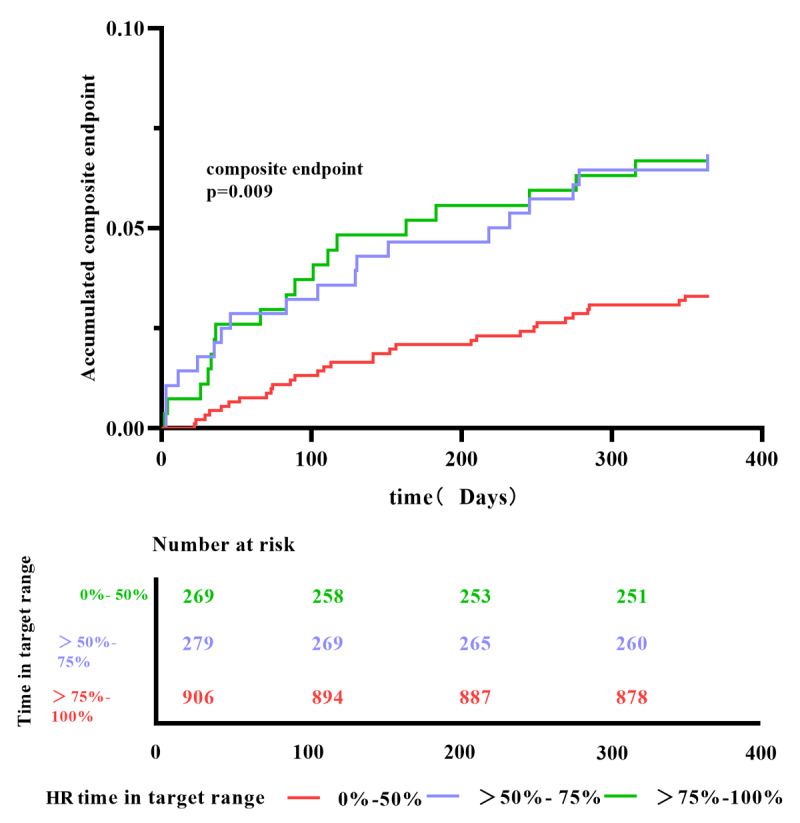
Kaplan–Meier survival curve from the 12-month follow-up under stratification according to heart rate in the target range.

As TTR increases, the risk of composite endpoint gradually decreases. Compared with the TTR-HR (>75%–100%) group, patients in TTR-HR (0–50%, >50%–75%) have the highest cumulative risk of composite endpoint (HR2.11, 95% CI: 1.19–3.74) (Figure 3), On Kaplan–Meier analysis for the composite endpoint, under stratification according to heart rate in the target range, patients with TTR-HR > 75%–100% had a better survival during the 12-month follow-up due to the maintaining the heart rate standard (Figure 4) (P = 0.009).

## 4 Discussion

The findings from our study offer a unique opportunity to gain insights into the association of heart rate control and adverse clinical outcomes among ACS patients following PCI. This study involved 1455 participants, digitally tracked for 12 months. Our results reveal that inadequate heart rate control, as indicated by TTR-HR, is significantly associated with a heightened risk of adverse clinical outcomes, including MACE, all-cause mortality, and cumulative adverse events. Through COX univariate and multivariate regression analysis, hypertension and TTR-HR were independently associated with adverse events in ACS patients. These findings underscore the significance of addressing both heart rate control and comorbidities, such as hypertension, in managing ACS patients. Additionally, our analysis revealed that patients with strict heart rate control, especially those between 75% and 100%, link with better outcomes. Stratified by the factors including age ≤70 years, non-smoking status, hypertension, heart failure, and non-diabetes, patients with TTR > 75% would in mitigating the risk of adverse clinical outcomes. This suggests that maintaining heart rate is crucial to obtain more clinical benefit in ACS patients following PCI.

Studies have shown that there is a semi-logarithmic inverse relationship between resting heart rate (HR) and life expectancy in mammals, and the questionable question is whether slowing heart rate can extend human lifespan (11). Among patients with chronic heart failure in the Systolic Heart failure treatment with the If inhibitor ivabradine trial, the risk of cardiovascular death or hospitalization due to heart failure is greater in patients with a high heart rate than in patients with a low heart rate, and may even be twice as high (12). Clinical registries indicate that a significant number of patients develop heart failure within the first 12 months following ACS, irrespective of whether they had pre-existing heart failure (13,14). While, an elevated heart rate (>75 beats per minute) significantly increases the hospitalization rate and mortality due to heart failure worsening among patients with heart failure following myocardial infarction (15,16,17). Hospitalization is crucial for these patients, but it can lead to a deterioration in quality of life, a heightened risk of mortality, and substantial medical costs (18,19,20). Enhancing the control of risk factors such as blood pressure, heart rate, and smoking in ACS patients after PCI can lower the incidence of adverse clinical outcomes (21). Therefore, it is particularly important to manage of postoperative risk factors after discharge.

In recent years, heart rate has garnered significant attention as a prognostic indicator and potentially modifiable risk factor, especially in coronary artery disease where therapeutic intervention may be needed (22). Prior investigations have elucidated that the adverse impact of an elevated heart rate may stem from an augmented myocardial oxygen consumption, ultimately resulting in ischemia and an enlargement of myocardial infarct size (23,24). An elevated heart rate not only exacerbates the progression of ischemia but also heightens the likelihood of ventricular arrhythmias. Furthermore, an increase in heart rate poses a greater risk for atherosclerosis and plaque rupture, leading to compromised diastolic filling and distal coronary perfusion (24). Notably, patients with coronary artery disease have demonstrated beneficial responses to the administration of rate-lowering medications(beta-blockers and ivabradine).

Compared to previous studies that have utilized a single heart rate measurement at a specific time point or the mean value over a given time period, our current study employs the concept of time in target range within the resting heart rate (25). Firstly, heart rate is a continuous, dynamic measurement, and a single value or average value is not sufficient to assess the objective state of blood pressure and its impact on clinical outcomes. Furthermore, the average of resting heart rate measurements over a period of time may fall within the target range, but the duration of reaching the expected level remains unknown (26). So, the time in target range within the resting heart rate in our study is more appropriate to evaluate the level of heart rate control. Heart rate has been shown to be associated with adverse outcomes in diverse populations with confirmed or suspected coronary artery disease (27). However, our study represents a novel contribution by investigating the correlation between post PCI target heart rate and long-term outcomes in a contemporary cohort of ACS patients who underwent percutaneous coronary revascularization. It emphasizes the importance of closely monitoring patients with heart failure during the post-PCI phase and cardiac rehabilitation, as heart rate target goals may serve as therapeutic targets warranting special attention. This research provides valuable insights for clinical practice and future interventions in this population.

It is noteworthy that although our study has shown a significant impact of heart rate on the prognosis of ACS patients following PCI, However, it is imperative to acknowledge the presence of certain limitations that warrant further enhancements and refinement in future investigations. (1) This study focused on ACS patients following PCI, excluding CABG patients, and cannot be extrapolated to other populations with ACS. Subsequent larger cohorts of different populations are needed to further improve our research. (2) Given the retrospective nature of this study, it is important to acknowledge that certain detailed patient information, including height, weight, GRACE score, and many details about PCI, will need well documented in future. Future studies could include prospective research and randomized controlled trials to further elucidate the impact of heart rate management strategies on the clinical outcomes of ACS patients after PCI. (3) Our study endpoint collected all-cause mortality, without cardiovascular mortality, limiting the prognosis analysis of heart rate on ACS patients. (4) The sample size in our study is relatively modest, which may contribute to statistically non-significant results in intergroup comparisons. Consequently, it is crucial to conduct additional research to validate and corroborate our findings.

## 5 Conclusions

In summary, our research findings emphasise the crucial role of heart rate control on adverse clinical outcomes in ACS patients following PCI. Optimising heart rate control with TTR may potentially provide benefits in improving overall patient outcomes. Future research includes prospective studies and randomized controlled trials to further elucidate the impact of heart rate management strategies on clinical outcomes of ACS patients following PCI. Our research findings have the potential to assist clinical physicians in promptly intervening in patients with abnormal heart rates, designing personalized treatment strategies, particularly for ACS individuals following PCI. This approach can effectively enhance patients’ quality of life, improve disease prognosis, and substantially mitigate the incidence of major adverse cardiac events and mortality.
